# A method combining the use of a mobile application and a dedicated pelvic positioner for acetabular cup insertion

**DOI:** 10.1186/s13018-022-03138-w

**Published:** 2022-05-03

**Authors:** Atsushi Kamimura, Makoto Enokida, Shinpei Enokida, Hideki Nagashima

**Affiliations:** Department of Orthopedic Surgery, Faculty of Medicine, Tottori University, 36-1 Nishi-cho, Yonago, Tottori 683-8504 Japan

**Keywords:** Total hip arthroplasty, Lateral decubitus position, Acetabular cup orientation, Application, Pelvic positioner

## Abstract

**Background:**

We developed a mobile device application and dedicated pelvic positioner with the aim of improving the acetabular cup placement accuracy in patients undergoing total hip arthroplasty (THA). The function of the application was to display the intra-operative cup angle. The accuracy and clinical usefulness of this combined method were verified through comparison with the conventional alignment guide method.

**Methods:**

In total, 60 patients who underwent cementless THA were included in this study. We compared the cup alignment when using this combined method with that when using the conventional alignment guide method. The absolute value error between the intra- and post-operative angles of inclination and anteversion of the cup was calculated.

**Results:**

The absolute value error of inclination was 2.4° ± 2.1° in the study group and 4.0° ± 3.3° in the control group (*P* = 0.107). The absolute value error of anteversion was 2.8° ± 2.6° in the study group and 7.4° ± 5.3° in the control group (*P* < 0.001).

**Conclusion:**

Using the application and pelvic positioner is simple and can be introduced at a low cost. The more accurate measurement of the intra-operative cup angle using this combined method has improved the cup insertion accuracy compared with that of the conventional alignment guide method.

## Background

The orientation of the acetabular cup in total hip arthroplasty (THA) affects the rate of dislocation, liner wear, range of motion, and long-term results [1, 2]. An alignment guide is usually used for the orientation of the acetabular cup. However, there are some reports of inaccuracy with this method [3–5]. It is said that a more limited safe zone should be set to improve treatment results [6, 7]. There are many opinions regarding introducing the navigation system as a more accurate cup placement method [8, 9]. Regardless of this, the navigation system introduction rate is still 14.1% in Japan [10]. The introduction rate of navigation systems is considered to be low primarily because it does not match the introduction and running costs.

There have been some reports on the usefulness of an inexpensive and simple surgical support system instead of an expensive navigation system [11, 12]. These surgical support systems have been introduced with the aim of clarifying the relationship between the reference pelvic plane and the intra-operative pelvic position or improving the ambiguity of the angle evaluation method when the cup insertion. To overcome both problems, we have developed a dedicated pelvic positioner that can reproduce the reference pelvic plane when placement the cup about the problem of intra-operative pelvic position and we created an application using the gyro sensor of the mobile device to deal with the ambiguity of the angle evaluation method when inserting the cup.

The purpose of this study was to verify the accuracy of the intra-operative angle measurement method that uses the pelvic positioner and the application of the mobile device together.

## Methods

In total, 60 patients (12 males, 48 females, average age of 63 [32–83] years) who underwent primary cementless THA between September 2014 and September 2017 at Tottori University Hospital were included in this study (Table 1). The control group consisted of 30 cases (5 males, 25 females, average age 59.3 [32–83] years) in which the alignment guide method was used from September 2014 to March 2016. The diseases of the control group were osteoarthritic hip in 20, femoral head osteonecrosis in 8, and rheumatoid arthritis in 2 cases. The study group included 30 patients (7 males, 23 females, average age of 67.6 [35–83] years) who underwent THA using our device from April 2016 to September 2017. The diseases of the study group were osteoarthritic hip in 25 cases and femoral head osteonecrosis in 5 cases. The iOS application named THA cup protractor (EGG CO., LTD. Yonago Japan) and the pelvic positioner (Nemoto Firm., Tokyo Japan) were jointly developed by us and each company as non-commercial products for this clinical study. Excluded subjects in this study were those for whom consent was not obtained, cases of primary THA in which a plate or support ring was used because of severe hip dysplasia, infectious hip arthritis, and revision THA.

**Table 1:** Patient demographic data

	Study group (30 hips)	Control group (30 hips)	*P* value
Patient age (years)	67.6	59.3	0.014
Gender (male: female)	7:23	5:25	0.519
Side (right, left)	14:16	15:15	0.796
BMI (kg/m^2^)	23.8	24.6	0.176
Diagnosis			
OA	25	20	0.084
ON	5	8	
RA	0	2	
Approach	Dall:15 Modified Watson–Jones:15	Dall:28 Modified Watson–Jones:2	< 0.001

BMI, body mass index, OA, osteoarthritis; ON, osteonecrosis; RA, rheumatoid arthritis

### Pre-operative planning

All surgeons (Y.K., A.K., S.E., and K.M.) are hip joint surgeons at our hospital. They made a pre-operative plan using 3D template software (the 3D Template ™ Japan Medical Materials, Osaka, Japan) and decided which implant model to use. Model selection was left to the discretion of each surgeon. The implants used in the control group were all AMS™ (Kyocera, Kyoto, Japan). In the study group, SCRUM™ (Kyocera, Kyoto, Japan) was used in 21, G7™ (Zimmer Biomet, Warsaw, Indiana) in 4, Continuum™ (Zimmer Biomet, Warsaw, Indiana) in 2, PINNACLE™ (DePuy Synthes, Warsaw, Indiana) in 2 cases and Trident™ (Stryker Orthopaedics, Mahwah, New Jersey) in 1 case. The cup model was selected based on the surgeon’s preference and affected by stem selection based on the shape of the medullary cavity of the femur. In all cases, computed tomography (CT) images were taken within one month before surgery. Digital Imaging and Communications in Medicine (DICOM) images were read with the 3D template software described above and used to determine the planned implant size and cup placement position and angle. The reference plane was set in the functional pelvic plane (FPP) [13]. The position of the cup was decided by each operator, but in principle, the radiographic inclination (RI) was 45° and the radiographic anteversion (RA) was 15°. In the study group, the center-edge angle was considered based on the 3D template of the pre-operative plan. We also planned to reduce the anteversion in patients with large posterior pelvic tilt. In the control group, the cup placement angles were 45° (inclination) and 15° (anteversion) with reference to the alignment guide in all cases.

### Surgical procedure

All operations were performed with the patient lateral decubitus position. In the control group, the Dall approach was selected in 28 cases and the modified Watson–Jones approach was selected in 2 cases. In the study group, the Dall approach was selected for 15 patients, whereas the modified Watson–Jones approach was selected for 15 patients. The approach was not randomised; however, the approach that each surgeon usually uses was selected. Since this study was also conducted in the introductory period of the minimally invasive surgery (MIS) approach, a high proportion of the study group underwent the modified Watson–Jones approach.

**Fig. 1:**
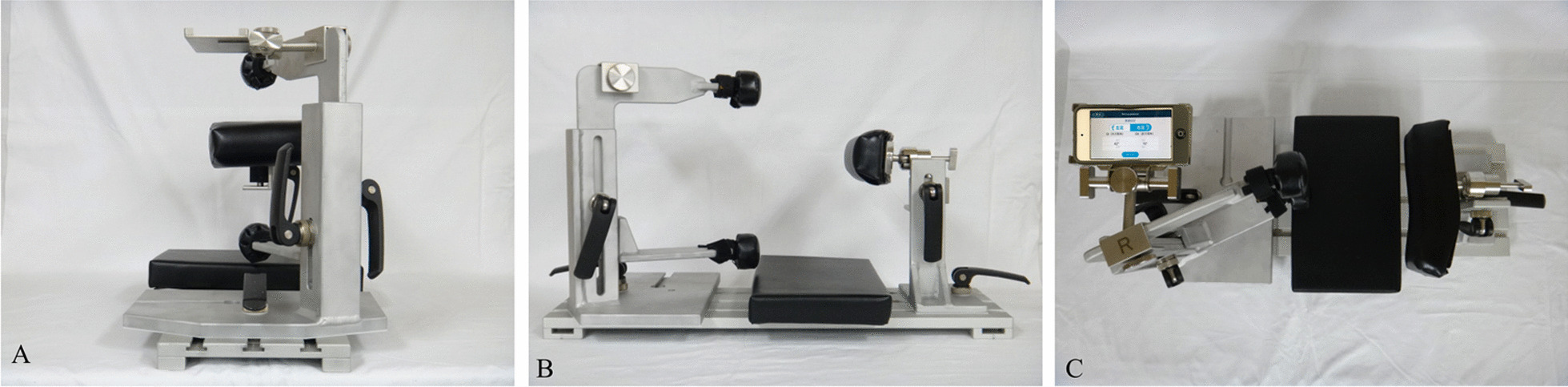
Pelvic positioner. Pelvic positioner has pads for fixing the ASIS on both sides and an accessory table for installing the iPod touch® at the time of reference. Pelvic positioner from anterior–posterior (**A**), cranio–caudal (**B**) and aerial view (**C**) is shown. ASIS, anterior superior iliac spine

**Fig. 2:**
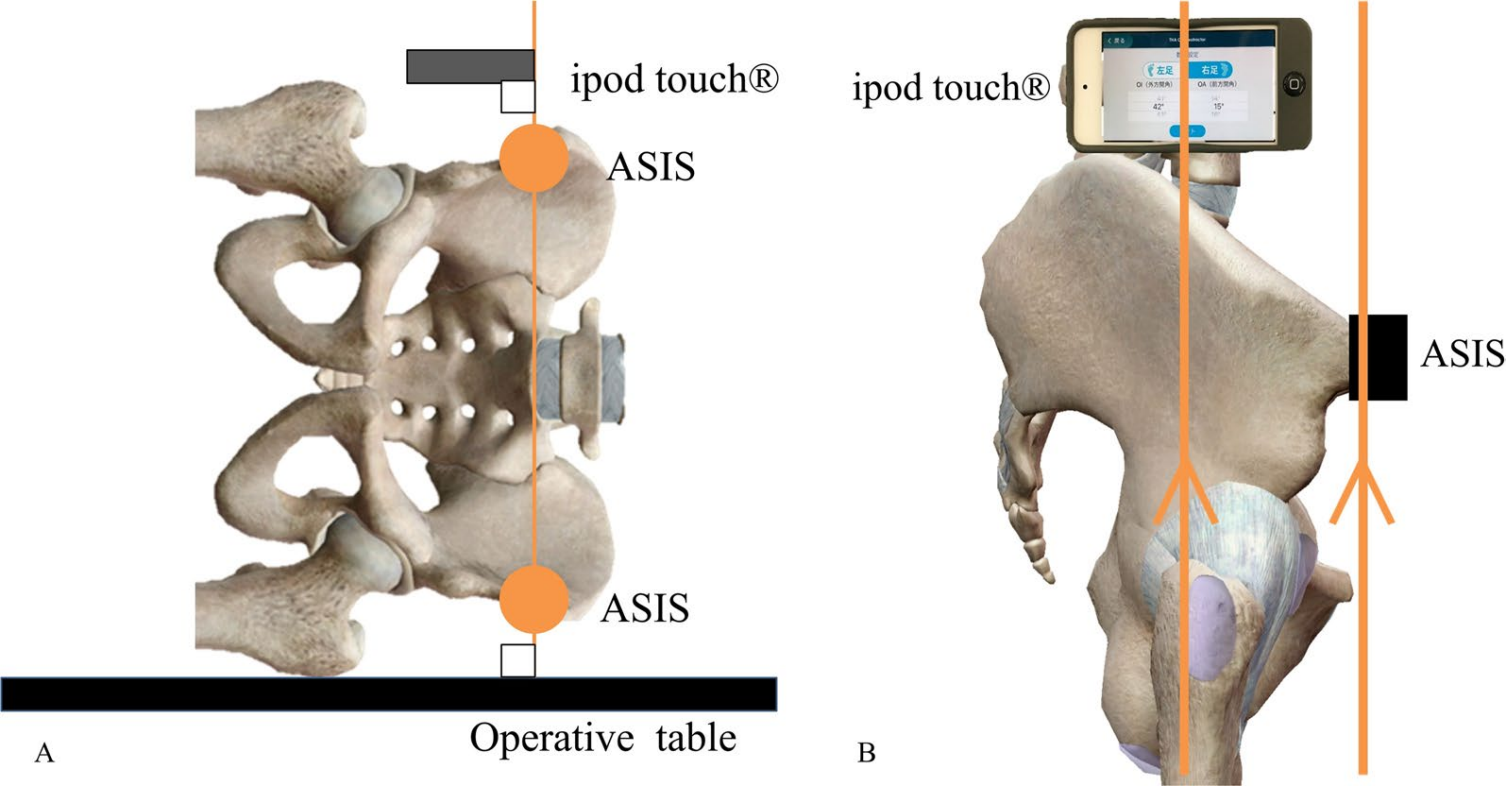
Fixing the pelvis using the pelvic positioner and setting the reference plane of the application. For setting the reference plane of the application on the accessory table: the reference line is parallel to the operating table and vertical to the line connecting the ASIS on both sides with the coronal plane (**A**) and parallel to the pelvic plane with the sagittal plane (**B**). ASIS, anterior superior iliac spine

**Fig. 3:**
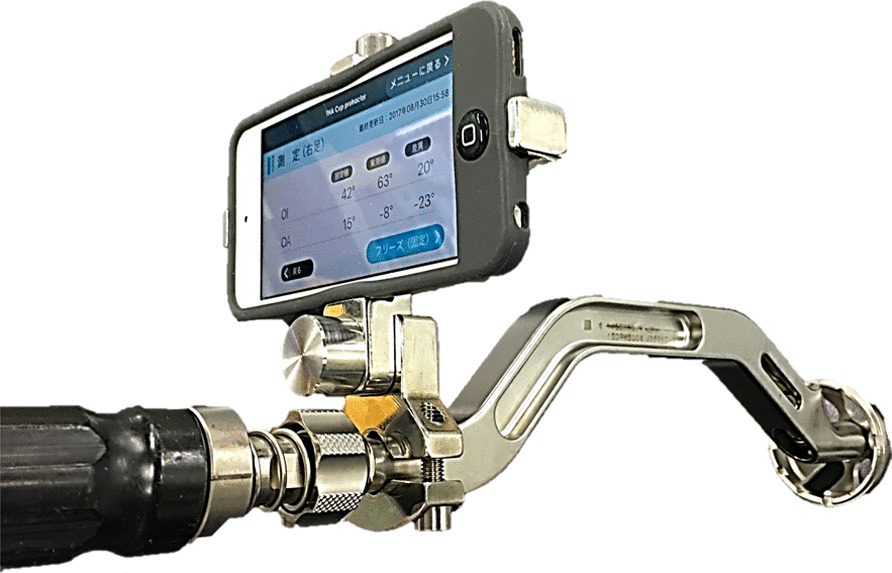
The iPod touch® connected to the cup impactor. The iPod touch® is used by connecting it to the cup impactor with a custom-made dedicated connector. The custom-made dedicated connector is designed such that the reference plane of the iPod touch® is parallel to the cup plane

**Fig. 4:**
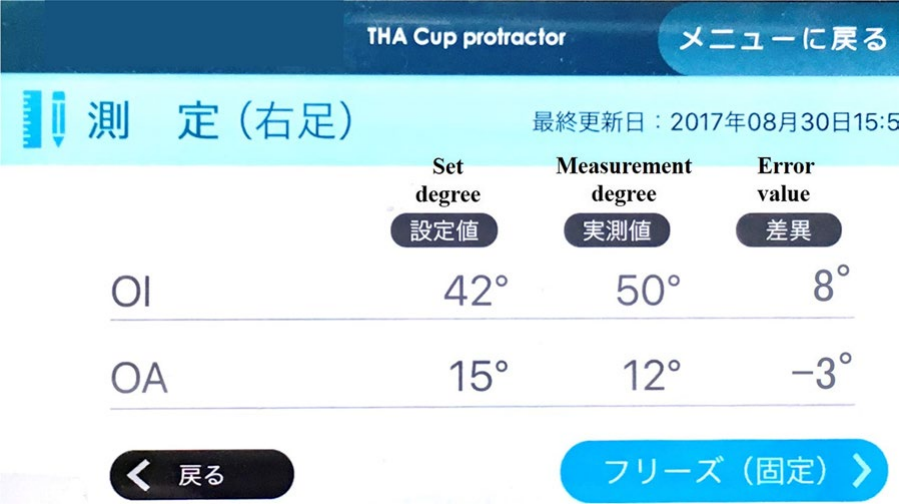
Screen of the application named THA cup protractor. The OI and OA set degree, measurement degree and error value are displayed simultaneously on the screen with an accuracy of 1°. The figure shows set degree OI 42° and OA 15°, measurement degree OI 50°, OA 12°, error value OI 8° and OA − 3°. OI, operative inclination; OA, operative anteversion

In the control group, the conventional alignment guide method was used [14]. The patient was placed in the lateral position, and the tilt of the operating table was adjusted so that the teardrop line was perpendicular to the floor and the obturator foramen was symmetrical. After confirming these under fluoroscopy, the symphysis pubis and sacrum were fixed from the front and back using the side plates. The cup alignment was visually adjusted with reference to the floor plane and the longitudinal axis plane of the body. In the study group, a dedicated pelvic positioner and iPod touch® having the THA cup protractor installed were used. The anterior superior iliac spine (ASIS) on both sides and the sacrum were fixed using a custom-made pelvic positioner. The pelvic positioner has pads for fixing the ASIS on both sides and an accessory table for installing the iPod touch® at the time of reference (Fig. 1). On fixing the ASIS on both sides to the center of the fixture pad, the ASIS gets aligned in the same plane, and the line connecting the ASIS on both sides becomes perpendicular to the operating table’s plane. By correcting the pelvic obliquity and rotation of the coronal plane, the intra-operative pelvic plane can be defined. The iPod touch® is covered with a sterile film. First, launch the application and enter the operative inclination (OI) and operative anteversion (OA) based on the pre-operative plan. Next, calibration is performed by installing the iPod touch® on the calibration table attached to the pelvic positioner. Because this table is designed to be parallel to the pelvic positioner in the coronal and sagittal planes, this calibration operation synchronises the pelvic positioner with the reference plane of the iPod touch® (Fig. 2). The iPod touch® is used by connecting it to the cup impactor with a custom-made dedicated connector. The custom-made dedicated connector is designed so that the reference plane of the iPod touch® is parallel to the cup plane (Fig. 3). Using the 3-axis gyro sensor of the iPod touch®, the amount of change in the coronal and sagittal plane angles is calculated from the calibrated reference plane. The set degree, measurement degree, and error value of OI and OA are displayed on the screen (Fig. 4).

### Radiographic assessments

In all cases, CT images were taken 2 weeks after surgery. The DICOM image was read with the 3D template software. The reference plane was set to the FPP. RI and RA were measured by matching cups of the same diameter on the software with surgically-placed cups. In this verification, inclination, and anteversion were unified with radiographic definitions [15], i.e., because the intra-operative application display angles, OI and OA are converted into RI and RA, Murray’s conversion formula [tan (RI) = tan (OI) / cos (OA), sin (RA) = sin (OA) × cos (OI)] was used. The achievement rates of the Lewinnek safe zone (inclination 40° ± 10°, anteversion 15° ± 10°) [5] were compared between the groups. For the study group, to verify the effect of the surgical approach on this method, the accuracy was compared and examined between the approaches.

### Sample size and statistical analysis

Power analyses were performed using G*Power 3.1.9.2 (Heinrich Heine, University of Dusseldorf, Dusseldorf, Germany). On conducting the pilot study with five cases in each group, it was found that the mean absolute value error of the cup placement angle was 2.9° ± 2.4° in the study group and 5.7° ± 4.1° in the control group. Based on the effect size of 0.83 obtained in the pilot study, a power calculation (α-error: 0.05, power: 0.8) was performed, which suggested that 50 patients (25 patients per group) would be required for conducting a trial with the absolute cup placement angle error as the endpoint.

The Mann–Whitney *U* test was used to compare the absolute cup installation angle error and age between groups. Student’s *t*-test was used for comparing body mass index (BMI) between groups. Pearson’s chi-square test was used to compare the achievement rates of gender, side, surgical approach, and Lewinnek safe zone. Fisher’s exact test was used to compare the achievement rates of diagnostics. For statistical analysis, IBM SPSS version 25 for Windows (SPSS Inc. Tokyo, Japan) was used. *P* < 0.05 was considered statistically significant.

## Results

The average age of patients was 67.6 years in the study group and 59.3 years in the control group (*P* = 0.014). The study group had the Dall approach for 15 hips and the modified Watson–Jones approach for 15 hips, whereas the control group had the Dall approach for 28 hips and the modified Watson–Jones approach for 2 hips. This shows a significant bias between the groups (*P* < 0.001) (Table 1).

**Fig. 5:**
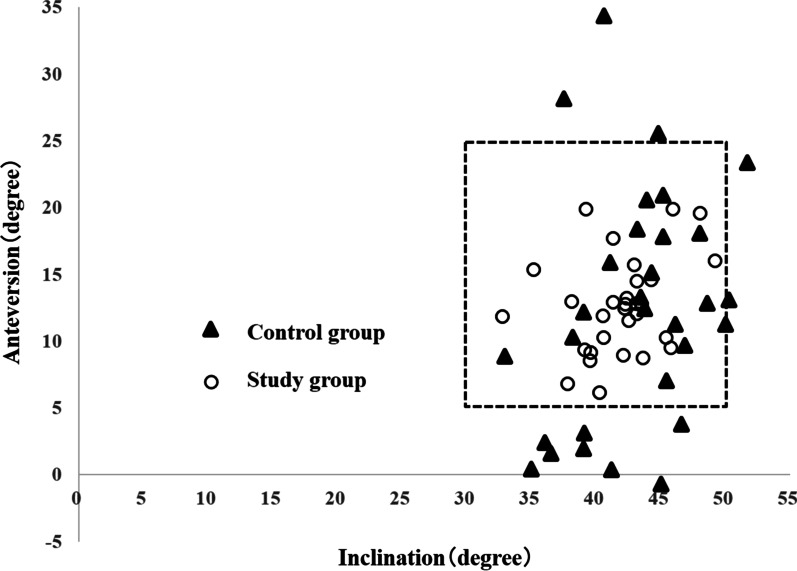
Scatter plot of cases achieving the Lewinnek safe zone. The cases in the dotted rectangle meet the requirements of the Lewinnek safe zone. The study group: thirty of thirty cases (100%) were within the conditions of the Lewinnek safe zone. The control group: 17 of 30 cases (56.7%) were within the safe zone

The absolute error between intra- and post-OI in the study group was 2.4° ± 2.1° and 4.0° ± 3.3° for the control group (*P* = 0.107). The anteversion was 2.8° ± 2.6° for the study group and 7.4° ± 5.3° for the control group (*P* < 0.001) (Table 2). As a result of verifying the post-operative cup placement, inclination was 41.8° ± 3.5° (32.8°–49.2°) and anteversion was 12.6° ± 3.6° (6.1°–19.9°) in the study group. In the control group, inclination was 43.0° ± 4.8° (32.9°–51.7°) and anteversion was 12.4° ± 8.9° (− 0.7°–34.4°), i.e., the achievement rate of Lewinnek safe zone (inclination 40° ± 10°, anteversion 15° ± 10°) [5] was 100% (30/30) for the study group and 56.7% (17/30) for the control group (*P* < 0.001) (Fig. 5).

**Table 2:** Absolute value error of intra-and post-operative cup placement angles

	Control group (30 hips)	Study group (30 hips)	*P* value
Intraoperative inclination (°)^a^	45	42.7 ± 3.1	
Postoperative inclination (°)^a^	43.0 ± 4.8	41.8 ± 3.5	
Absolute value error of inclination (°)^a^	4.0 ± 3.3	2.4 ± 2.1	0.107^b^
Intraoperative anteversion (°)^a^	15	11.0 ± 4.4	
Postoperative anteversion (°)^a^	12.4 ± 8.9	12.6 ± 3.6	
Absolute value error of anteversion (°)^a^	7.4 ± 5.3	2.8 ± 2.6	< 0.001^b^

^a^Mean ± standard deviation

^b^Mann–Whitney *U* test

Regarding the effect of the surgical approach on the cup placement accuracy using this method, the absolute error between intra- and post-OI in the study group was 2.3° ± 2.1° for the Dall approach and 2.5° ± 2.2° for the modified Watson–Jones approach (*P* = 0.819). The absolute error between intra- and post-OA was 2.2° ± 1.6° with the Dall approach and 3.4° ± 3.3° with the modified Watson–Jones approach (*P* = 0.430). No statistically significant difference was observed between the groups. This suggests that the surgical approach does not impact our method.

## Discussion

The cup placement accuracy using our method was 2.4° ± 2.1° for inclination and 2.8° ± 2.6° for anteversion. The method using the alignment guide reportedly deviates from the safe zone of Lewinnek in many cases [16]. Some previous reports have investigated accuracy of image-free navigation systems and CT-based navigation systems. Accuracy of CT-based navigation systems is reported as 1.2°–3.2° for inclination and 1.0°–3.3° for anteversion [8, 17–19]. Accuracy of image-free navigation systems is reported as 2.9°–3.6° for inclination and 4.2°–6.7° for anteversion [17, 20–22] (Table 3). Although the result obtained using our method was inferior to that obtained using the CT-based navigation system, it was comparable to the result obtained using image-free navigation. These results indicate that this method has practicality that can demonstrate clinically reliable accuracy. Although not evaluated in this study, this method may be useful in performing revision THA that requires orientation.

**Table 3:** Accuracy of imageless and CT-based navigation systems reported in the literature

	Inclination (°) Absolute value error	Anteversion (°) Absolute value error	Type	Navigation system
Kalteis (30 hips)	3.0 ± 2.6	3.3 ± 2.3	CT-based	The Vector Vision hip 3.0
Iwana (117 hips)	1.8 ± 1.6	1.2 ± 1.1	CT-based	Stryker CT-Hip System V1.0–29
Nakahara (49 hips)	1.2 ± 1.3	1.0 ± 0.5	CT-based	Stryker Navigation System2
Tetsunaga (30 hips)	3.2 ± 2.4	3.0 ± 2.5	CT-based	The Vector Vision Hip CT-based version 3.5.2
Kalteis (30 hips)	2.9 ± 2.2	4.2 ± 3.3	Image-free	The Vector Vision hip 3.0
Ybinger (37 hips)	3.5 ± 4.4	6.5 ± 7.3	Image-free	The PiGalileo THR, Plus
Lass (62 hips)	3.2 ± 2.4	6.5 ± 3.7	Image-free	The Navitrack
Takeda (118 hips)	3.6 ± 2.6	6.7 ± 3.6	Image-free	The Orthopilot THA Pro
Current study (30 hips)	2.4 ± 2.1	2.8 ± 2.6		

In 2012, Peters reported an intra-operative angle measurement method using the accelerometer and camera function of the iPhone for improving the accuracy without using the navigation system [12]. In that method, inclination is measured using an application that uses an accelerometer, whereas anteversion is measured using an application that displays a protractor with a camera function. This method does not consider the movement of the pelvis during surgery and assumes that the pelvic plane is always in the ideal position.

The alignment guide method, which is based on the floor plane and the longitudinal axis plane of the body, is susceptible to intra-operative pelvic movement [23]. Kanazawa reports that the pelvis tilts in each of the sagittal, axial, and coronal planes during surgery [24]. Compared with Peters’ method, our method can correct intra-operative pelvic movement with the help of the pelvic positioner.

Since the THA cup protractor is a simple digital angle measuring application, it cannot follow intra-operative pelvic movement like the navigation system. Therefore, when measuring the placement angle, it is necessary to confirm that the ASIS is in the center of the pelvic positioner fixture. If there is a deviation, it is necessary to return the positional relationship between ASIS and pelvic positioner to the state at the time of set-up.

Navigation systems generally require pins to be inserted into the pelvic to fix the navigation tracker. Therefore, it requires invasion of the patient and additional operative time. In comparison, our method uses the pelvic positioner as a reference plane; thereby making patient invasion unnecessary which is also an advantage of our method.

It has been reported that the surgical approach affects the cup placement accuracy. The minimally invasive surgery (MIS) approach has the disadvantage that anatomical recognition is difficult because of the small field of view. Also, the cup placement accuracy is inferior because it is easily affected by intra-operative pelvic movement. Therefore, it is recommended that the navigation system be used in this approach [25]. The modified Watson–Jones approach, which is a MIS approach, has the advantage of maintaining hip abduction muscle strength and posterior stability. However, there are many variations in the cup placement position, such as a significantly larger inclination than the posterior approach [26]. In this study, there was no significant difference in the cup placement accuracy between the modified Watson–Jones approach and the Dall approach. This result suggests that our method ensures high cup placement accuracy irrespective of the approach used.

This study has limitation. It was not randomised, however, the patients’ demographic factors were unlikely to have affected the results because the two groups were comparable in terms of gender, BMI, and underlying disease.

## Conclusion

We developed an application named THA cup protractor and a pelvic positioner and used them to report the cup placement accuracy in patients who underwent cementless THA. Its accuracy is superior to that of the alignment guide method. Although there is room for improvement, it is excellent inconvenience and cost performance. It is considered a good system for consideration in non-navigation THA.

## Data Availability

The datasets analyzed during the current study are available from the corresponding author on reasonable request.

## Abbreviations

THA: Total hip arthroplasty
APP: Anatomical pelvic plane
OI: Operative inclination
OA: Operative anteversion
FPP: Functional pelvic plane
RI: Radiographic inclination
RA: Radiographic anteversion
BMI: Body mass index
ASIS: Anterior superior iliac spine

## References

[1] Grammatopoulos G, Thomas GE, Pandit H, Beard DJ, Gill HS, Murray DW (2015). The effect of orientation of the acetabular component on outcome following total hip arthroplasty with small diameter hard-on-soft bearings. Bone Joint J.

[2] Biedermann R, Tonin A, Krismer M, Rachbauer F, Eibl G, Stöckl B (2005). Reducing the risk of dislocation after total hip arthroplasty: the effect of orientation of the acetabular component. J Bone Joint Surg Br.

[3] Digioia AM, Jaramaz B, Plakseychuk AY, Moody JE, Nikou C, Labarca RS (2002). Comparison of a mechanical acetabular alignment guide with computer placement of the socket. J Arthroplasty.

[4] Hassan DM, Johnston GH, Dust WN, Watson G, Dolovich AT (1998). Accuracy of intraoperative assessment of acetabular prosthesis placement. J Arthroplasty.

[5] Lewinnek GE, Lewis JL, Tarr R, Compere CL, Zimmerman JR (1978). Dislocations after total hip-replacement arthroplasties. J Bone Joint Surg Am.

[6] Danoff JR, Bobman JT, Cunn G, Murtaugh T, Gorroochurn P, Geller JA (2016). Redefining the acetabular component safe zone for posterior approach total hip arthroplasty. J Arthroplasty.

[7] Widmer KH, Zurfluh B (2004). Compliant positioning of total hip components for optimal range of motion. J Orthop Res.

[8] Iwana D, Nakamura N, Miki H, Kitada M, Hananouchi T, Sugano N (2013). Accuracy of angle and position of the cup using computed tomography-based navigation systems in total hip arthroplasty. Comput Aided Surg.

[9] Ryan JA, Jamali AA, Bargar WL (2010). Accuracy of computer navigation for acetabular component placement in THA. Clin Orthop Relat Res.

[10] THA Japanese Registry 2018. In: Japanese Society for Replacement Arthroplasty. https://jsra.info/pdf/2017.pdf.

[11] Iwakiri K, Kobayashi A, Ohta Y, Minoda Y, Takaoka K, Nakamura H (2017). Efficacy of a pelvic lateral positioner with a mechanical cup navigator based on the anatomical pelvic plane in total hip arthroplasty. J Arthroplasty.

[12] Peters FM, Greeff R, Goldstein N, Frey CT (2012). Improving acetabular cup orientation in total hip arthroplasty by using smartphone technology. J Arthroplasty.

[13] Nishihara S, Sugano N, Nishii T, Ohzono K, Yoshikawa H (2003). Measurements of pelvic flexion angle using three-dimensional computed tomography. Clin Orthop Relat Res.

[14] Kamimura A, Kishimoto Y, Okano T (2013). Adjust of pelvic inclination in total hip arthroplasty using preoperative fluoroscope. Cent Jpn J Orthop Surg Traumatol.

[15] Murray DW (1993). The definition and measurement of acetabular orientation. J Bone Joint Surg Br.

[16] Saxler G, Marx A, Vandevelde D, Langlotz U, Tannast M, Wiese M (2004). The accuracy of free-hand cup positioning—a CT based measurement of cup placement in 105 total hip arthroplasties. Int Orthop.

[17] Kalteis T, Handel M, Bäthis H, Perlick L, Tingart M, Grifka J (2006). Imageless navigation for insertion of the acetabular component in total hip arthroplasty: is it as accurate as CT-based navigation?. J Bone Joint Surg Br.

[18] Nakahara I, Kyo T, Kuroda Y, Miki H (2018). Effect of improved navigation performance on the accuracy of implant placement in total hip arthroplasty with a CT-based navigation system. J Artif Organs.

[19] Tetsunaga T, Yamada K, Tetsunaga T, Furumatsu T, Sanki T, Kawamura Y (2020). Comparison of the accuracy of CT- and accelerometer-based navigation systems for cup orientation in total hip arthroplasty. Hip Int.

[20] Ybinger T, Kumpan W, Hoffart HE, Muschalik B, Bullmann W, Zweymüller K (2007). Accuracy of navigation-assisted acetabular component positioning studied by computed tomography measurements: methods and results. J Arthroplasty.

[21] Lass R, Kubista B, Olischar B, Frantal S, Windhager R, Giurea A (2014). Total hip arthroplasty using imageless computer-assisted hip navigation: a prospective randomized study. J Arthroplasty.

[22] Takeda Y, Fukunishi S, Nishio S, Fujihara Y, Yoshiya S (2017). Accuracy of component orientation and leg length adjustment in total hip arthroplasty using image-free navigation. Open Orthop J.

[23] Grammatopoulos G, Pandit HG, da Assunção R, Taylor A, McLardy-Smith P, De Smet KA (2014). Pelvic position and movement during hip replacement. Bone Joint J.

[24] Kanazawa M, Nakashima Y, Ohishi M, Hamai S, Motomura G, Yamamoto T (2016). Pelvic tilt and movement during total hip arthroplasty in the lateral decubitus position. Mod Rheumatol.

[25] DiGioia AM, Plakseychuk AY, Levison TJ, Jaramaz B (2003). Mini-incision technique for total hip arthroplasty with navigation. J Arthroplasty.

[26] Laffosse JM, Accadbled F, Molinier F, Chiron P, Hocine B, Puget J (2008). Anterolateral mini-invasive versus posterior mini-invasive approach for primary total hip replacement. Comparison of exposure and implant positioning. Arch Orthop Trauma Surg.

